# Sensitive detection and quantification of SARS-CoV-2 by multiplex droplet digital RT-PCR

**DOI:** 10.1007/s10096-020-04076-3

**Published:** 2020-10-26

**Authors:** Remco de Kock, Mieke Baselmans, Volkher Scharnhorst, Birgit Deiman

**Affiliations:** 1 Clinical Laboratory, Catharina Hospital Eindhoven, Eindhoven, The Netherlands; 2 Institute for Complex Molecular Systems and Department of Biomedical Engineering, Laboratory of Chemical Biology, Eindhoven University of Technology, Eindhoven, The Netherlands; 3 Expert Center Clinical Chemistry Eindhoven, Eindhoven, The Netherlands

**Keywords:** SARS-CoV-2, ddPCR, Multiplex, RT-PCR, Quantification, Monitoring

## Abstract

The purpose of this study is to develop a one-step droplet digital RT-PCR (RT-ddPCR) multiplex assay that allows for sensitive quantification of SARS-CoV-2 RNA with respect to human-derived RNA and could be used for screening and monitoring of Covid-19 patients. A one-step RT-ddPCR multiplex assay was developed for simultaneous detection of SARS-CoV-2 *E, RdRp* and *N* viral RNA, and human *Rpp30* DNA and *GUSB* mRNA, for internal nucleic acid (NA) extraction and RT-PCR control. Dilution series of viral RNA transcripts were prepared in water and total NA extract of Covid-19-negative patients. As reference assay, an *E-GUSB* duplex RT-PCR was used. *GUSB* mRNA detection was used to set validity criteria to assure viral RNA and RT-PCR assay quality and to enable quantification of SARS-CoV-2 RNA. In a background of at least 100 *GUSB* mRNA copies, 5 copies of viral RNA are reliably detectable and 10 copies viral RNA copies are reliably quantifiable. It was found that assay sensitivity of the RT-ddPCR was not affected by the total NA background while assay sensitivity of the gold standard RT-PCR assay is drastically decreased when SARS-CoV-2 copies were detected in a background of total NA extract compared with water. The present study describes a robust and sensitive one-step ddRT-PCR multiplex assay for reliable quantification of SARS-CoV-2 RNA. By determining the fractional abundance of viral RNA with respect to a human housekeeping gene, viral loads from different samples can be compared, what could be used to investigate the infectiveness and to monitor Covid-19 patients.

## Introduction

The outbreak of Covid-19 caused by SARS-CoV-2 has spread worldwide. Up to now, over 35 million confirmed cases have been reported. The USA, Brazil, and India are the most affected countries with the highest mortality rates due to Covid-19 [1].

The gold standard for the detection of SARS-CoV-2 is based on real-time reverse transcriptase PCR (RT-PCR). In the Netherlands, the detection of the envelope (*E)* gene, followed by confirmatory testing of the RNA-dependent RNA polymerase *(RdRp)* gene, is recommended [2]. Another approach is to detect the nucleocapsid *(N)* gene and to use an open reading frame 1a/b *(ORF1b)* gene or *E* gene assay as a confirmatory test [3]. In addition, to improve assay sensitivity, other studies have been focusing on the detection of *N*, *E*, or *ORF1b* using droplet digital polymerase chain reaction (ddPCR) [4–7].

As the RNA genome of SARS-CoV-2 mutates during virus replication, false negative results could be obtained due to the loss of primer or probe binding [8, 9]. By using multiplex assays, targeting various SARS-CoV-2 genes, the chance of missing a positive sample in this way is reduced.

The amount and quality of viral RNA in the patient sample is an important factor for reliable virus detection. Also, most assays do not include a patient-derived internal RNA control for the reverse transcriptase step, which is a critical step in viral RNA detection. The inclusion of a patient-derived internal RNA control would enable for quality control of the (viral) RNA in the patient sample and the RT-PCR.

Finally, quantification of the SARS-CoV-2 viral load becomes more and more important to distinguish positive patients who are infectious from positive patients with only residual viral RNA who are probably not infectious anymore (viral shedding) [10] and for monitoring Covid-19 patients during treatment.

The goal of this study is to develop a sensitive one-step droplet digital RT-PCR (RT-ddPCR) multiplex assay for simultaneous detection of multiple SARS-CoV-2 genes *N (N1+N2)*, *E*, and *RdRp,* including the detection of patient-derived mRNA of a housekeeping gene to assure sample and assay quality and to enable quantification of viral RNA.

## Methods

### Samples

As reference standard, the Wuhan Coronavirus 2019 *E* gene control (EVAg, European Virus Archive Global, France), an in vitro transcript (100,000 copies/mL), was used directly for amplification. Additionally, transcript RNA of the *E, N, ORF1ab, RdRp,* and *S* gene (all 200,000 copies/mL), extracted from the Exact Diagnostics SARS-CoV-2 Standard (EDx, Exact Diagnostics, Texas, USA) using the MP24 Total NA isolation Kit, on the MagNA Pure 24 system (Roche Diagnostics, Rotkreuz, Switzerland), was used for amplification.

Dilution series of the reference standards were prepared in nuclease-free water and in remnant total nucleic acid (NA) MagNaPure 24 extract from nasopharyngeal swabs of Covid-19-negative patients, to obtain an input of 5–500 copies per reaction for the EVAg control and 2.5–1000 copies per reaction for the EDx control.

### One-step reverse transcriptase real-time PCR

The one-step reverse transcriptase reaction for the detection of the *E* gene was performed in a 25 μL reaction volume as described previously [2]. The assay was also performed in a 10 μL reaction volume, consisting of 5 μL 2× reaction buffer, 0.16 μL of a 50 mM magnesium sulfate solution and 0.40 μL of SuperScript™ III RT/Platinum™ Taq Mix (all provided by the SuperScript™ III One-Step RT-PCR with Platinum™ Taq Polymerase, Invitrogen, USA), 400 nM forward primer, 400 nM reverse primer, 200 nM probe (primers and probe provided by TIB MolBiol, Berlin, Germany), 0.4 μg of nonacetylated bovine serum albumin (Ultrapure™ BSA, Invitrogen, USA), and 2 μL of RNA.

The *E-GUSB* duplex RT-PCR was performed in a 25 μL and 10 μL reaction volume as described above, with the addition of 1.25 μL and 0.5 μL of the *GUSB* Gene Expression assay (Bio-Rad Laboratories, Hercules, CA), respectively.

All assays were performed on the LightCycler 480 II system (Roche Diagnostics) using the following cycling conditions: 10 min at 55 °C for reverse transcription, followed by 3 min at 95 °C, continuing with 15 s at 95 °C, and 30 s at 58 °C.

### One-step reverse transcriptase droplet digital PCR multiplex

The ddPCR multiplex assay allows for the simultaneous detection of *E*, *RdRp*, *N (N1+N2)*, *Rpp30*, and *GUSB*. For each assay, a reaction mixture of 22 μL was prepared with 17 μL amplification mix and 5 μL RNA extract. The amplification mix, based on the one-step RT-ddPCR advanced kit for probes (Bio-Rad Laboratories), consisted of 5.5 μL Supermix, 2.2 μL reverse transcriptase, and 1.1 μL 300 mM DTT (Bio-Rad Laboratories). The primers and probes for the detection of *E* and SARS-CoV-2 specific *RdRp* were described previously [2] except for the *E* reverse primer, as using this primer, false positive reactions were obtained. For *E*, 450 nmol forward primer [2], 450 nmol in-house reverse primer (5′-GGTTTTACAAGACTCACGTTAACA-3′) (TIB MolBiol), and 250 nmol HEX-labeled and 250 nmol FAM-labeled probe [2] (Integrated DNA technologies, Coralville, USA) were added; for *RdRp*, 900 nmol forward and reverse primers and 250 nmol probe [2] were added; for *N*, 1.0 μL 2019-nCov CDC ddPCR Triplex Probe assay (Bio-Rad Laboratories) was added and 1.0 μL of *GUSB* assay (Bio-Rad Laboratories) was added.

The QX200 Droplet Digital PCR System (Bio-Rad Laboratories) was used for the quantification of SARS-CoV-2 RNA. For droplet generation, 20 μL reaction mix was used and the droplets were transferred to a 96-well plate. Samples were amplified in the C1000 Touch Thermal Cycler (Bio-Rad Laboratories) according to following protocol: 50 °C for 60 min (reverse transcription), 95 °C for 10 min (enzyme activation), 40 cycles of 95 °C for 30 s (denaturation) and 55 °C for 60 s (annealing), and 98 °C for 10 min (enzyme deactivation). Data were analyzed using QuantaSoft version 1.7.4.0917 (Bio-Rad Laboratories).

## Results

**Fig. 1:**
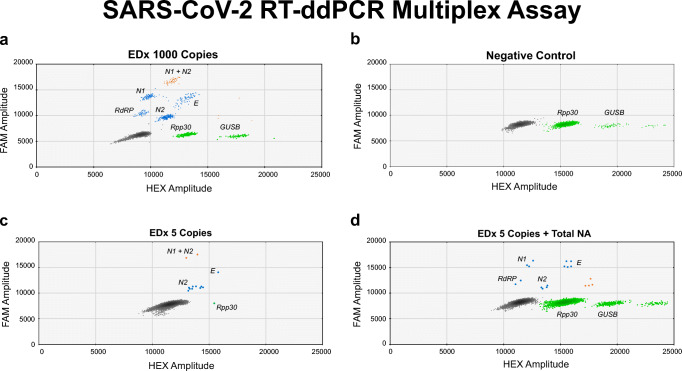
Two-dimensional scatterplots of the SARS-CoV-2 RT-ddPCR multiplex assay targeting *N*, *E*, and *RdRp* (FAM). *Rpp30* is included as internal DNA control and *GUSB* as internal reverse transcriptase control (HEX). **a** Undiluted EDx standard with 1000 SARS-CoV-2 copies per reaction (*n* = 1). **b** Total NA extract from nasopharyngeal swabs from Covid-19-negative patients. **c** EDx standard, diluted in water, with 5 SARS-CoV-2 copies per reaction (*n* = 4). **d** EDx standard, diluted in total NA extract from Covid-19-negative patients, with 5 SARS-CoV-2 copies per reaction (*n* = 4)

Recently, a commercial RT-ddPCR assay, targeting two highly conserved regions of the SARS-CoV-2 *N* gene became available (2019-nCoV CDC ddPCR Triplex Probe assay, Bio-Rad Laboratories). This assay also includes an internal control for the detection of patient-derived *Rpp30* as internal DNA control for the PCR step. Based on this assay, a multiplex one-step RT-ddPCR test was developed for the detection of the *E*, *RdRp*, and *N (N1+N2)* gene*.* In addition, primers and probes for the detection of patient-derived *GUSB* mRNA were added as reverse transcriptase control. The multiplex was tested on the EDx reference standard, including transcripts of *E, RdRp*, and *N* and human genomic total nucleic acid (NA). For each SARS-CoV-2 target, a distinct cluster was identified in the two-dimensional scatterplot (Fig. 1a). For a reproducibility analysis, 5 replicates of the EDx standard were tested and quantified, showing that the SARS-CoV-2 concentrations were all in the same order of magnitude and in agreement with the input used (Table 1).

**Table 1:** Validation samples tested with the SARS-CoV-2 RT-ddPCR multiplex assay

Sample	SARS-CoV-2 (copies/reaction)	*Rpp30* (copies/reaction)	*GUSB* (copies/reaction)	FA (%)
Positive control 1	1018 (946–1090)	488 (438–536)	116 (92–140)	90
Positive control 2	806 (732–882)	308 (262–356)	62 (42–84)	93
Positive control 3	868 (796–942)	472 (420–526)	64 (46–86)	93
Positive control 4	848 (776–922)	430 (378–482)	74 (54–98)	92
Positive control 5	896 (816–978)	452 (396–510)	110 (84–140)	90
Negative control 1	ND	512 (450–574)	370 (316–422)	
Negative control 2	ND	1580 (1460–1700)	14 (6–30)	
Negative control 3	ND	1800 (1700–1920)	108 (84–134)	
Negative control 4	ND	11,660 (11,320–12,000)	466 (406–526)	
Negative control 5	ND	5000 (4820–5200)	128 (100–158)	

The undiluted EDx standard was used as positive control and total NA extract from remnant nasopharyngeal swabs from Covid-19-negative patients as negative control

*ND* not detected, *FA* fractional abundance of viral RNA calculated with respect to the *GUSB* concentration

### Assay sensitivity

**Fig. 2:**
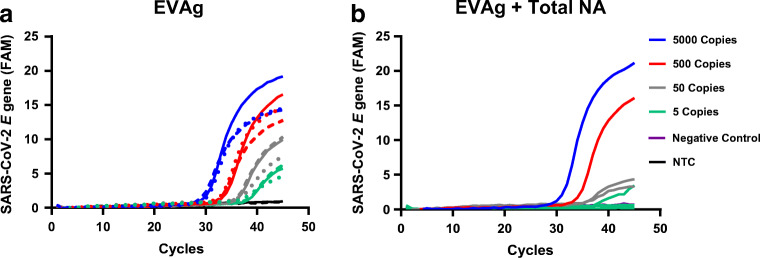
Real-time amplification curves of the *E* gene RT-PCR reference assay tested on the EVAg standard. The reference assay was described previously [2]. **a** EVAg dilution series with 5–5000 SARS-CoV-2 copies/reaction diluted in water. The continuous lines represent the reference assay in 25 μL reaction volume (*n* = 1), the interrupted lines represent the reference assay in 10 μL reaction volume (*n* = 1), and the dotted lines represent the *E-GUSB* duplex RT-PCR assay (*n* = 1). **b** EVAg dilution series in total NA extract from a Covid-19-negative patient tested with the RT-PCR assay. For 500–5000 copies/reaction (*n* = 1), for 50 copies/reaction (*n* = 2), for 5 copies/reaction (*n* = 4)

To determine assay sensitivity of the SARS-CoV-2 RT-ddPCR multiplex assay, the previously described one-step reverse transcriptase RT-PCR targeting the *E* gene [2] was set up and used as reference assay. To ensure the correct performance of this reference assay, a dilution series of the EVAg standard was tested, showing that the *E* target could be detected down to an input of 5 copies per reaction (Fig. 2a), which is in agreement with the performance of this assay as described previously [2].

To reflect a more realistic clinical setting, a comparable dilution series of the EVAg standard was prepared in total NA extract obtained from nasopharyngeal swabs of Covid-19-negative patients. Strikingly, for the EVAg standard diluted in NA extract, 5 SARS-CoV-2 copies per reaction was no longer detectable and only 1 out of 4 reactions was tested positive, suggesting loss of sensitivity when compared to the EVAg standard diluted in water (Fig. 2a and b).

As this reference assay does not include the detection of a patient-derived internal control, a duplex reaction was developed combining the *E* gene reference assay [2] with the *GUSB* assay (Bio-Rad Laboratories). The sensitivity of the *E-GUSB* duplex RT-PCR was tested, using the previously described dilution series of the EVAg standard in water (Fig. 2a). Results show that using the *E-GUSB* duplex RT-PCR, an input of 5 copies per reaction is still detectable, suggesting no loss of assay sensitivity compared with the *E* gene reference assay [2].

**Fig. 3:**
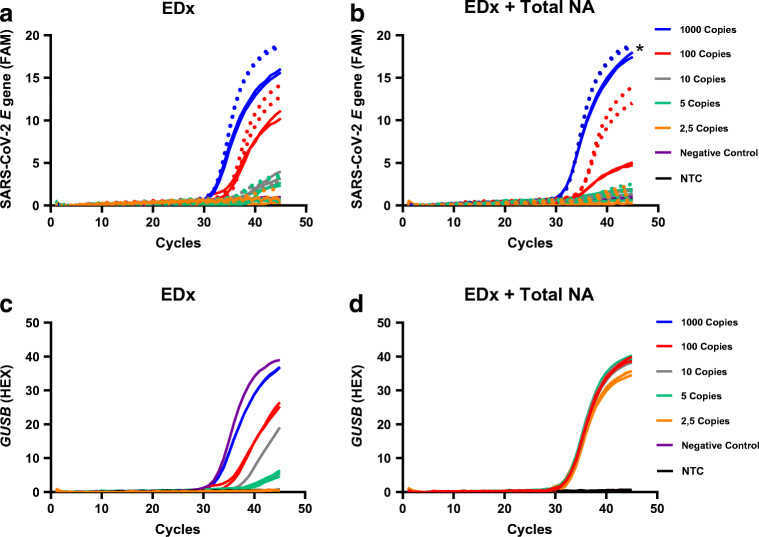
Real-time amplification curves of the *E* gene reference and the *E-GUSB* duplex assay tested on the EDx standard. **a**, **c** EDx dilution series with 2.5–1000 SARS-CoV-2 copies/reaction diluted in water. **b**, **d** EDx dilution series in total NA extract of a Covid-19-negative patient. The dotted lines represent the *E* gene reference assay [2]. *EDx dilution in water. For 10–1000 copies/reaction (*n* = 2), 2.5–5 copies/reaction (*n* = 4)

Next, both the *E* gene reference assay [2] and the *E-GUSB* duplex assay were tested using a dilution series in water of the EDx standard, including *GUSB* mRNA. Again, no loss of sensitivity was observed when comparing the performance of the *E* gene reference assay and the *E-GUSB* duplex assay (Fig. 3a; Table 2). For both assays, using an input of 10 SARS-CoV-2 EDx copies per reaction, 100% was tested positive (*n* = 2), while using an input of 5 SARS-CoV-2 EDx copies per reaction, 50% tested positive (*n* = 4; Fig. 3a and c; Table 2). EDx copies are not identical to EVAg copies as the EVAg standard is used directly in amplification, while the EDx standard requires extraction, like patient samples, during which some RNA will be lost. Again, when using dilution series of the EDx standard prepared in total NA extract from Covid-19-negative patients, a serious loss of sensitivity was observed for both the *E* gene reference assay and the *E-GUSB* duplex assay, as 5 and 10 SARS-CoV-2 EDx copies per reaction were no longer reliably detectable (Fig. 3b and d; Table 2). The *E-GUSB* duplex RT-PCR was further used as reference assay to investigate the assay performance of the SARS-CoV-2 RT-ddPCR multiplex assay.

**Table 2:** EDx dilution series tested using the SARS-CoV-2 RT-PCR *E* gene reference*, *E-GUSB* duplex, and RT-ddPCR multiplex assay

EDx standard (Cp/reaction)	RT-PCR (Ct value)					RT-ddPCR (copies/reaction)				
	*E* Simplex		*E-GUSB* Duplex							
	SARS-CoV-2		SARS-CoV-2		*GUSB*	SARS-CoV-2		*Rpp30*	*GUSB*	FA (%)
	Water	Total NA	Water	Total NA		Water	Total NA			
1000	31.7	NA	31.3	NA	NA	1098 (1004–1192)	NA	NA	NA	NA
1000	31.7	NA	31.6	NA	NA	1002 (916–1090)	NA	NA	NA	NA
100	34.2	34.2	34.4	33.1	32.2	108 (80–140)	94 (70–124)	4531 (4338–4726)	155 (123–192)	38
100	34.7	34.4	34.6	33.6	32.2	114 (86–150)	96 (74–124)	4343 (4164–4523)	115 (89–145)	45
10	34.1	ND	36.3	ND	32.2	20 (10–34)	5.6 (1.4–14.8)	5028 (4830–5229)	160 (128–196)	3
10	34.5	ND	36.8	ND	32.1	12 (4–24)	18 (8–32)	4990 (4788–5194)	152 (120–188)	10
5	ND	ND	ND	ND	31.9	9 (2.6–21)	9 (3.2–19.6)	5254 (5054–5457)	112 (86–142)	7
5	ND	ND	37.3	ND	32.0	2.4 (0.2–11.8)	3.2 (0.4–10)	4942 (4761–5125)	129 (103–159)	2
5	35.2	35.6	36.9	ND	32.0	7.6 (2.2–17.8)	11.6 (4.8–22.4)	4818 (4635–5002)	124 (98–154)	9
5	35.2	ND	ND	ND	32.0	5.6 (1.4–14.8)	10.2 (3.6–22.4)	5256 (5041–5472)	148 (117–186)	6
2.5	ND	ND	ND	ND	32.1	ND	ND	4820 (4640–5020)	122 (96–154)	
2.5	ND	ND	ND	ND	32.2	ND	ND	5160 (4960–5380)	106 (80–138)	
2.5	35.3	ND	ND	ND	32.0	ND	3.4 (0.6–11)	4840 (4660–5040)	112 (86–142)	3
2.5	ND	35.6	ND	ND	32.3	ND	ND	4860 (4660–5060)	112 (86–144)	

*NA* not applicable, *ND* not detected, *FA* fractional abundance of viral RNA calculated with respect to the *GUSB* concentration

*Gold standard assay [2]

**Fig. 4:**
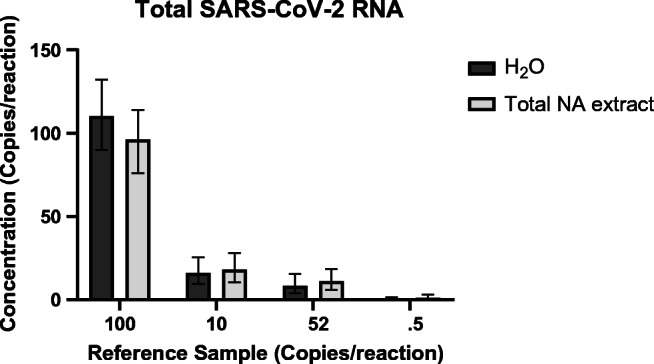
Dilution series of the EDx standard diluted in water or total NA extract of a Covid-19-negative nasopharyngeal swab tested with the SARS-CoV-2 RT-ddPCR multiplex assay. For 10–100 copies/reaction (*n* = 2), 2.5–5 copies/reaction (*n* = 4)

The same EDx dilution series, in water and in total NA extract of Covid-19-negative patients, were used to investigate the sensitivity of the SARS-CoV-2 RT-ddPCR multiplex assay. Using 5 SARS-CoV-2 copies per reaction of the EDx standard diluted in water, SARS-CoV-2 RNA could be detected (*n* = 4; Fig. 1c; Table 2), which is comparable with the assay sensitivity of the *E-GUSB* duplex RT-PCR. Fortunately, when using the EDx standard diluted in total NA extract from Covid-19-negative patients, the SARS-CoV-2 multiplex RT-ddPCR assay could again detect 5 SARS-CoV-2 copies per reaction (*n* = 4; Fig. 1d; Table 2). For each dilution, in water or total NA extract, the detected SARS-CoV-2 concentration was in the same order of magnitude (Fig. 4; Table 2), indicating that the sensitivity of the RT-ddPCR multiplex assay is not affected by the total NA extract. In addition, in the presence of total NA extract, the SARS-CoV-2 multiplex RT-ddPCR assay is approximately 10-fold more sensitive than the *E-GUSB* duplex RT-PCR assay. The limit of detection (LoD) of the SARS-CoV-2 multiplex RT-ddPCR assay is estimated to be 5 copies per reaction.

### Quantification of viral RNA

To correct for the differences in RNA yield and quality obtained during sample collection and differences in efficiency of the RT-ddPCR, quantification of the viral load was performed relative to the patient-derived *GUSB* mRNA (fractional abundance, FA). For the undiluted EDx standard, a reproducible FA of approximately 90% was obtained, showing the robustness of the FA determination (Table 1).

For the EDx standard dilution series in total NA extract, obtained from Covid-19-negative patients, a *GUSB* mRNA concentration of at least 100 copies per reaction was detected (Table 2). In this background of total NA, down to 5 SARS-CoV-2 copies per reaction can be detected, but the limit of quantification (LoQ) is estimated to be 10 copies per reaction, as the FA obtained with 5 or 10 SARS-CoV-2 copies per reaction overlaps (Table 2).

For 5 Covid-19-negative patient samples, the *GUSB* mRNA concentration was determined individually (Fig. 1b; Table 1). Remarkably, for one sample, a *GUSB* mRNA concentration of just 14 copies per reaction, so far below 100 copies per reaction, was found, implying poor sample collection or poor reverse transcription that could have resulted in a false negative result.

## Discussion

A novel RT-ddPCR multiplex assay was developed targeting three different genes of the SARS-CoV-2 virus. As the SARS-CoV-2 genome evolves rapidly [8, 9], it is of interest to screen multiple targets simultaneously to avoid possible mismatches of primers and probes, which could lead to false negative results [11]. The SARS-CoV-2 RT-ddPCR multiplex assay also includes a patient-derived NA extraction control and a reverse transcriptase control to ensure adequate sample and assay quality required for reliable virus detection.

While reverse transcriptase RT-PCR is still the gold standard, the findings in the present study indicate that the assay sensitivity of the RT-PCR assay is reduced due to background NA from the patient sample. By contrast, the sensitivity of the RT-ddPCR multiplex assay was not affected by background NA and is more sensitive than the gold standard reverse transcriptase RT-PCR [4–7] in the clinical setting.

As ddPCR enables absolute quantification, not only the viral RNA can be quantified but also the *GUSB* mRNA, which can be used to set validity criteria and to ensure reliable analysis, as false negative results may occur due to poor sample quality as a result of inappropriate sample collection, handling, or transportation [12]. For the SARS-CoV-2 RT-ddPCR developed in this study, 5 copies of viral RNA are reliably detectable and 10 copies viral RNA copies are reliably quantifiable in a background of at least 100 *GUSB* mRNA copies.

By quantification of SARS-CoV-2 relative to patient-derived *GUSB* mRNA, the fractional abundance of the viral loads of different samples can be compared. This could be used to gain insights in the relation between viral load and infectivity, which is at this moment unclear [13–16].

Together, this study presents a sensitive one-step RT-ddPCR multiplex assay that allows for reliable detection and quantification of SARS-CoV-2 viral RNA with respect to patient-derived mRNA of a house-keeping gene, what could be used for triage and enables disease monitoring of Covid-19 patients.

## Data Availability

Upon request.

## References

[1] COVID-19 Dashboard by the Center for Systems Science and Engineering (CSSE) at Johns Hopkins University (JHU). https://coronavirus.jhu.edu/map.html. Accessed 13 Oct 2020

[2] Corman VM et al (2020) Detection of 2019 novel coronavirus (2019-nCoV) by real-time RT-PCR. Euro Surveill Bull Eur Sur Mal Transm Eur Commun Dis Bull 25(3). 10.2807/1560-7917.ES.2020.25.3.2000045 10.2807/1560-7917.ES.2020.25.3.2000045 PMC6988269 31992387

[3] Chu DKW (2020). Molecular diagnosis of a novel coronavirus (2019-nCoV) causing an outbreak of pneumonia. Clin Chem.

[4] Suo T et al (2020) ddPCR: a more accurate tool for SARS-CoV-2 detection in low viral load specimens. Emerg Microbes Infect 1–30. 10.1080/22221751.2020.1772678 10.1080/22221751.2020.1772678 PMC7448897 32438868

[5] Yu F et al (2020) Quantitative detection and viral load analysis of SARS-CoV-2 in infected patients. Clin Infect Dis Off Publ Infect Dis Soc Am. 10.1093/cid/ciaa345 10.1093/cid/ciaa345 PMC7184442 32221523

[6] Dong L et al (2020) Highly accurate and sensitive diagnostic detection of SARS-CoV-2 by digital PCR’. Public and Global Health, preprint. 10.1101/2020.03.14.20036129 10.1016/j.talanta.2020.121726 PMC7588801 33379001

[7] Falzone L (2020). Sensitivity assessment of droplet digital PCR for SARS-CoV-2 detection. Int J Mol Med.

[8] Khailany RA, Safdar M, en Ozaslan M (2020) Genomic characterization of a novel SARS-CoV-2. Gene Rep 100682. 10.1016/j.genrep.2020.100682 10.1016/j.genrep.2020.100682 PMC7161481 32300673

[9] Pachetti M (2020). Emerging SARS-CoV-2 mutation hot spots include a novel RNA-dependent-RNA polymerase variant. J Transl Med.

[10] Younes N (2020). Challenges in laboratory diagnosis of the novel coronavirus SARS-CoV-2. Viruses.

[11] Muenchhoff M (2020). Multicentre comparison of quantitative PCR-based assays to detect SARS-CoV-2, Germany, March 2020. Euro Surveill Bull Eur Sur Mal Transm Eur Commun Dis Bull.

[12] Tahamtan A, Ardebili A (2020). Real-time RT-PCR in COVID-19 detection: issues affecting the results. Expert Rev Mol Diagn.

[13] Widders A, Broom A, Broom J (2020). SARS-CoV-2: the viral shedding vs infectivity dilemma. Infect Dis Health.

[14] La Scola B (2020). Viral RNA load as determined by cell culture as a management tool for discharge of SARS-CoV-2 patients from infectious disease wards. Eur J Clin Microbiol Infect Dis Off Publ Eur Soc Clin Microbiol.

[15] Zou L (2020). SARS-CoV-2 viral load in upper respiratory specimens of infected patients. N Engl J Med.

[16] Zhou R (2020). Viral dynamics in asymptomatic patients with COVID-19. Int J Infect Dis IJID Off Publ Int Soc Infect Dis.

